# Controlling flowering of *Medicago sativa* (alfalfa) by inducing dominant mutations

**DOI:** 10.1111/jipb.13186

**Published:** 2022-01-18

**Authors:** Maurizio Junior Chiurazzi, Anton Frisgaard Nørrevang, Pedro García, Pablo D. Cerdán, Michael Palmgren, Stephan Wenkel

**Affiliations:** ^1^ NovoCrops Center University of Copenhagen Thorvaldsensvej 40 Frederiksberg C 1871 Denmark; ^2^ Copenhagen Plant Science Centre University of Copenhagen Thorvaldsensvej 40 Frederiksberg C 1871 Denmark; ^3^ Department of Plant and Environmental Sciences University of Copenhagen Thorvaldsensvej 40 Frederiksberg C 1871 Denmark; ^4^ Fundación Instituto Leloir IIBBA‐CONICET Avenida Patricias Argentinas 435 Buenos Aires 1405 Argentina

**Keywords:** flowering time, genome‐engineering, *Medicago sativa*, microProtein, microRNA

## Abstract

Breeding plants with polyploid genomes is challenging because functional redundancy hampers the identification of loss‐of‐function mutants. *Medicago sativa* is tetraploid and obligate outcrossing, which together with inbreeding depression complicates traditional breeding approaches in obtaining plants with a stable growth habit. Inducing dominant mutations would provide an alternative strategy to introduce domestication traits in plants with high gene redundancy. Here we describe two complementary strategies to induce dominant mutations in the *M. sativa* genome and how they can be relevant in the control of flowering time. First, we outline a genome‐engineering strategy that harnesses the use of microProteins as developmental regulators. MicroProteins are small proteins that appeared during genome evolution from genes encoding larger proteins. Genome‐engineering allows us to retrace evolution and create microProtein‐coding genes *de novo*. Second, we provide an inventory of genes regulated by microRNAs that control plant development. Making respective gene transcripts microRNA‐resistant by inducing point mutations can uncouple microRNA regulation. Finally, we investigated the recently published genomes of *M. sativa* and provide an inventory of breeding targets, some of which, when mutated, are likely to result in dominant traits.

## Graphical abstract

Flowering time is a crucial trait for alfalfa improvement, but alfalfa is tetraploid and non‐selfable, hindering breeding. An investigation of the alfalfa genome yielded strategies to induce dominant mutations that would delay fl owering, removing the need for homozygosity.

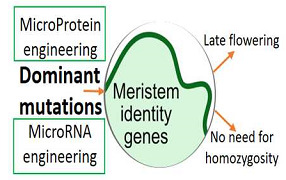

## *MEDICAGO SATIVA*, **ITS USE IN AGRICULTURE, BENEFITS, AND CURRENT CHALLENGES**

M*edicago sativa* (hereafter “alfalfa”), commonly known as alfalfa or lucerne, is a perennial forage legume typically used for hay, silage and pasture production (Hawkins and Yu, 2018). It has been named “the Queen of Forages,” because of its high yield, nutritional value, and protein content, and high resilience in adverse environments (Russelle, 2001). Additionally, its good palatability for animals makes it the most used forage and one of the most widely grown crops in the world. Besides these attributes listed above, alfalfa shows additional interesting characteristics: being a legume, it can fix atmospheric nitrogen, reducing the need for chemical fertilization, for which reason it is included strategically in crop cycles to naturally enrich the soil nitrogen levels. As a deep‐rooting plant, alfalfa is more resistant to drought when compared to other forages and aids in improving the physical properties of the soil (Putnam et al., 2001). All these characteristics make it an economically valuable crop for sustainable agriculture.

Given these interesting attributes, one would expect alfalfa to be at the center of attention in breeding programs. However, breeding of alfalfa has proven to be difficult (Abberton and Marshall, 2005). First, alfalfa is an autotetraploid plant, which means that its chromosome complement consists of four copies of a single genome due to doubling of an ancestral chromosome complement. Given that each homolog can pair with any of the other three, segregation proportions are different and more difficult to follow during improvement programs. Accordingly, traditional breeding is complicated in alfalfa, and has been very much depending on phenotypic selection, known to be a time‐demanding process (Burton, 1974). In addition, breeding of alfalfa is further complicated by a strong inbreeding depression (Li and Brummer, 2012).

Despite these difficulties, there are good margins for improvement of many alfalfa traits, from biomass production to the digestibility of the forage. Despite its high protein content, when compared to other forages, alfalfa shows a relatively low digestibility, due to its high lignin (Knudsen, 1997) and low tannin contents (McMahon et al., 2000). The amount of lignin is dependent on the foliage and the leaf‐to‐stem ratio. A high leaf‐to‐stem‐ratio results in more leaf biomass and less stems resulting in lower lignin amounts and improved digestibility (Sheaffer et al., 2000). In this perspective, flowering time is an important trait because it is directly related to yield and forage quality (Jung and Muller, 2009). The correlations between flowering and yield have been investigated in depth in other crops such as cereals (Distelfeld et al., 2009; Shrestha et al., 2014; Liu et al., 2020) but there is still a lack of knowledge in herbaceous perennials such as alfalfa. Nevertheless, recent evidence has started to shed light on genes that control flowering time in alfalfa, that can be targeted to extend the duration of the vegetative phase, which is strongly correlated with yield and forage quality (Lorenzo et al., 2019). Plants that flower late produce more biomass because most of the resources and photosynthates are reallocated to the inflorescence during the transition to flowering. Inversely, early flowering plants show decreased yields and lower forage quality and digestibility (Wang et al., 2013).

The above‐mentioned challenges for traditional breeding suggest that a biotechnology‐focused approach may prove more effective in generating improved alfalfa varieties in less time. Efforts in alfalfa improvement using genetic engineering approaches have recently been used to improve digestibility by reducing the lignin content (Barros et al., 2019). This review will focus on regulation of flowering time and on the possibility to extend the vegetative phase using biotechnological approaches. We will review how alfalfa flowering time and the length of the vegetative phase are to be considered key and central traits in alfalfa improvement. After evaluating different traits of interest and assessing the current knowledge and the currently available alfalfa genomic resources, we will propose candidate target genes and strategies for genome‐engineering approaches likely to result in dominant phenotypes.

## IDENTIFYING MOLECULAR BREEDING TARGETS FOR REGULATION OF FLOWERING TIME IN ALFALFA

We consider *CONSTANS* (*CO), APETALA2* (*AP2), SQUAMOSA PROMOTER BINDING PROTEIN‐LIKE* (*SPL*), *miR172* and *miR156* and *TEOSINTE BRANCHED1‐CYCLOIDEA‐AND‐PCF* (*TCP*) to be important targets for directed improvement of alfalfa as these genes and miRNAs are well‐known to control development in model plants, including phase transition, flowering time, flower development, leaf and organ size, and shade sensitivity (Chen, 2004; Wu et al., 2009; Shim et al., 2017; Zheng et al., 2019). Their functions have been well studied in Arabidopsis and our aim has been to define possible alfalfa orthologs of these genes.

With the recent availability of alfalfa genomic data (Chen et al., 2020; Shen et al., 2020) it has become easier to propose possible strategies for targeting genes with a biotech approach and, once a putative target gene has been identified, it can now be addressed in a more straight‐forward way (Lei et al., 2017; Hawkins and Yu, 2018; Adhikari et al., 2019; Hrbackova et al., 2020). We investigated the currently available alfalfa genomic resources and searched for the aforementioned targets.

We started by mining the recently published alfalfa genome (Chen et al., 2020) and compared the sequences of selected target genes to their homologs from *Medicago truncatula, Glycine max*, and Arabidopsis *thaliana*. Based on current knowledge on their roles and on phylogenetic analyses, we collected what we consider to be some of the most interesting breeding targets in alfalfa (Table 1). Phylogenetic trees based on the identified sequences can aid the identification of genes to be modified in genome‐engineering approaches such as two of the main strategies that we are proposing in this article.

**Table 1:** Potential breeding targets in *Medicago sativa* (alfalfa)

Trait	Gene name in Arabidopsis	Gene Name in M. sativa	Chromosome coordinates	Gene IDs	*M. truncatula* identity (%)	*A. thaliana* identity (%)
Flower and seed development, flowering time regulation	*APETALA 2*	*msAP2La*	chr5.2:9079992. .9083100	ms.gene041052	95.58	69.39
		*msAP2Lb*	chr5.2:9000798. .9003906	ms.gene56970	95.58	69.39
		*msAP2Lc*	Chr5.4:10064967. .10068089;,	ms.gene010316	95.58	52.80
Flower and seed development, flowering time regulation	*APETALA 2*	*msAP2Ld*	chr8.1:26612058. .26614958	ms.gene011791	95.98	52.15
		*msAP2Le*	chr8.2:25356149. .25359054	ms.gene56806	96.17	51.97
		*msAP2Lf*	Chr8.4:26136457. .26139362;;	ms.gene99769	95.98	51.95
Flowering time regulation	*CONSTANS*	*msCOL1a*	Chr7.4:81972764. .81975540	ms.gene44781	91.56	49.87
		*msCOL1b*	chr7.1:78680354. .78683137	ms.gene022048	91.81	49.25
		*msCOL1c*	chr7.3:80151355. .80154132	ms.gene75965	91.07	49.24
		*msCOL1d*	chr7.2:79559250. .79562476	ms.gene62915	91.07	49.62
Possible Flowering time regulation, branch length	*CONSTANS LIKE 15*	*msCOL10a*	chr7.1:21630593. .21634748	ms.gene033677	93.78	53.51
		*msCOL10b*	chr7.4:23501187. .23504944	ms.gene72598	93.03	53.51
		*msCOL10c*	chr7.2:23825851. .23829601	ms.gene54974	93.53	53.51
Flowering time Main regulator	*FLOWERING LOCUS T*	*msFT1a*	Chr7.2:22710331. .22712062	ms.gene51913	98.30	71.10
		*msFT1b*	chr7.1:20022840:20023363	ms.gene41686	98.27	71.12
		*msFT1c*	chr7.3:23632608:23634335	ms.gene51950	98.30	71.10
		*msFT1d*	chr7.4:22261511:22265508	ms.gene51911	62.50	62.07
*Regulation of flowering time and yield*	*Micro RNA 156*	*msMir156a*	chr1.1 6243475. .6244230	No Gene ID	97.87	64.58
		*msMir156b*	chr1.4 6555508. .6556261	No Gene ID	97.87	64.58
		*msMir156c*	chr1.3 6401226. .6401972	No Gene ID	97.87	63.27
*Leaf and shoot development, flowering time*	*SQUAMOSA BINDING PROTEIN 3*	*msSPL3a*	chr4.3 24686603. .24691012	No Gene ID	95.83	68.00
		*msSPL3b*	chr4.1 21416346. .21420754	No Gene ID	95.14	67.00
		*msSPL3c*	chr4.4 24283233. .24287651	No Gene ID	95.27	67.00
		*msSPL3d*	chr4.2 22572983. . 22576318	No Gene ID	90.54	67.00
Regulation of Flowering time and leaf development	*TEOSINTE BRANCHED1; CYCLOIDEA; PROLIFERATING CELL FACTOR*	*msTCP3a*	Chr2.4:20312360. .20313268;	ms.gene059738	89.77	86.75
		*msTCP3b*	chr2.2:16493699. .16494601	ms.gene060651	92.00	86.75
Regulation of flowering time, secondary wall thickness and leaf development	*TEOSINTE BRANCHED1; CYCLOIDEA; PROLIFERATING CELL FACTOR*	*msTCP4a*	Chr8.2:46469547. .46470848	ms.gene032256	95.22	48.26
		*msTCP4b*	chr8.3:46640186. .46641481	ms.gene007917	91.08	47.07
		*msTCP4c*	chr8.4:46996525:46997817	ms.gene34255	91.99	47.07
		*msTCP4d*	chr8.1:52038072:52039379	ms.gene36024	93.09	45.96

Based on their known function in Arabidopsis and some of the roles shown in alfalfa, the central breeding targets discussed in this review are shown. The trait of interest that the target genes would control is shown in the table, together with the gene names, both in Arabidopsis and in alfalfa (using the names that the genes were assigned in the conducted phylogenetic analyses shown in Figures S1–S3), the alfalfa chromosome coordinates and gene Ids (based on Chen et al., 2020) and the percentages of sequence identity of alfalfa, with both *M. truncatula* and Arabidopsis.

## MICROPROTEINS AND MIRNAS AS PROMISING TARGETS TO INDUCE DOMINANT PHENOTYPES

Considering the above‐detailed characteristics and limitations of alfalfa breeding, we propose a strategy based on the generation of dominant mutations to uncouple microRNA regulation and a CRISPR‐induced deletion approach to generate *de novo* microProteins. Such dominant mutations would generate a stable phenotype already in the heterozygote state, alleviating the need for homozygosity and allowing outcrossing of alfalfa.

### MicroProteins and the CONSTANS family

MicroProteins are small, usually single‐domain proteins that are sequence‐related to larger, often multidomain proteins. They can heterodimerize with their targets displaying a compatible protein‐protein interaction domain and engage them in protein complexes. MicroProtein‐dependent regulation has been shown to be an intrinsic negative regulatory feedback of different biological processes, not only in plants (Eguen et al., 2015). MiP1a/b‐type microProteins contain a B‐Box domain, are related to the CONSTANS transcription factor and were shown to modulate flowering and photomorphogenesis in Arabidopsis (Graeff et al., 2016; Yadav et al., 2019). MiP1a/b‐type microProteins also have an additional TOPLESS‐interaction domain. TOPLESS is a transcription co‐repressor protein having a role in the auxin signaling (Szemenyei et al., 2008). The miP1a/b microProteins interact with TOPLESS and engage COSTANS in a trimeric repressor complex.

**Figure 1:**
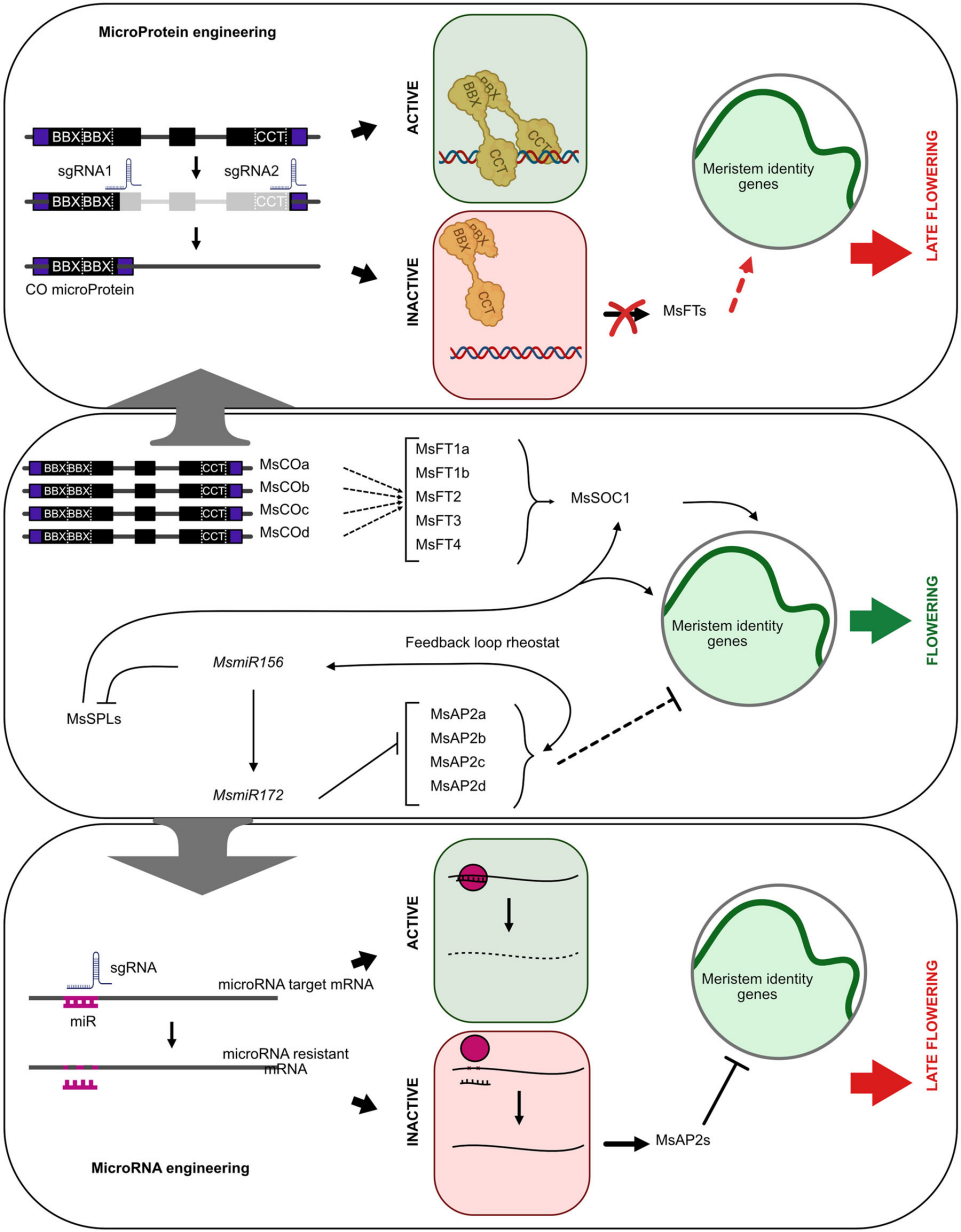
Hypothetical flowering pathways in *Medicago sativa* (alfalfa) and the proposed genome engineering strategies for the induction of dominant mutations resulting in delayed flowering **Top panel:** The microProtein strategy. CONSTANS/CONSTANS‐LIKE transcription factors act by forming homo‐/heterodimeric protein complexes through their B‐Box (BBX) domains while the CCT‐domain has DNA‐binding functions. The activity of CONSTANS/CONSTANS‐LIKE proteins can be modulated by expressing BBX‐type microProteins. Genome‐engineering can be used to convert *CONSTANS/CONSTANS‐LIKE* genes into BBX microProteins. Shown is a hypothetical CO/COL gene with exons in black and UTRs in purple. SgRNAs can be designed that anneal after the BBX and CCT‐domain respectively resulting in the chromosomal loss depicted in grey. After NHEJ, the *CO/COL* gene has been converted into a gene now encoding a BBX microProtein. **Middle panel:** The hypothetical flowering pathways in alfalfa based on the main breeding targets discussed in this review. **Bottom panel:** The miR‐binding site mutation strategy. A CRISPR‐mediated mutation of the *miR172* binding sites of the *AFP2* family members would result in lack of *miR172* binding and in *AFP2s* being able to downregulate flowering activator genes.

It has been shown that microProteins can be generated in different ways: directly as a small transcript from a single gene (*trans*‐microProteins) or from alternative transcription events (e.g., splicing, alternative transcription start site or polyadenylation site choices; referred to as *cis*‐microProteins). Interestingly, it was also shown that microProteins can be synthetically engineered by truncating parts of a transcription unit, thereby generating smaller versions of the full‐length transcript. In the latter case, the truncated protein can heterodimerize with the full‐length proteins produced by homologous gene family members. The synthetic microProtein can interact and thereby inhibit these related proteins in a dominant‐negative fashion (Figure 1). This has been shown in Arabidopsis, where parts of the coding sequence of the *AFP2* gene that encodes a NINJA‐domain protein were deleted by using a (CRISPR)/Cas‐9 approach (Hong et al., 2020). NINJA proteins function as negative regulators of jasmonic acid (JA) responses. The NINJA‐related microProtein, LITTLE NINJA (LNJ), was first discovered in *Brachypodium* as a factor affecting plant size and bushiness by interacting with NINJA and thus changing its jasmonic acid regulation (Hong et al., 2020). These findings show that engineering microProteins from individual genes is a possibility that has the potential to establish novel regulatory feedback loops.

The CONSTANS/CO‐LIKE gene family is suitable for the generation of a dominant microProtein feedback loop since truncated variants have been shown to affect flowering in other crop plants (Eguen et al., 2020). The B‐Box zinc finger transcription factor *CONSTANS* (*CO*) is well known in Arabidopsis as the major regulator of the photoperiod pathway (Putterill et al., 1995). CO activates another central flowering regulator, the *FLOWERING LOCUS T* (*FT*) gene (florigen), expressed in the phloem (An et al., 2004). The FT protein is then transported to the meristem, where it induces flowering (Corbesier et al., 2007; Tamaki et al., 2007). Since its initial discovery, many *CONSTANS‐like* (*COL*) orthologs have been identified in Arabidopsis and other plant species. The CO/COL gene family was previously characterized in many legume species such as *Pisum sativum* (Hecht et al., 2005), *Lotus japonicus* (Hecht et al., 2005), *G. max* (Wu et al., 2014), and *M. truncatula* (Wong et al., 2014) but so far not in alfalfa. In alfalfa's closest diploid relative, *M. truncatula*, despite CO orthologues being present in the genome, so far no CO genes were found to be actively playing a role in flowering, suggesting that the flowering pathway might differ from that of model plants (Hecht et al., 2005; Jaudal et al., 2016). Studies have reported that *MtCOL* mutants from COL group I do not display any difference in flowering time, and complementation experiments in a *col2* mutant Arabidopsis background also could not revert the late flowering phenotype (Wong et al., 2014). Similarly, transient expression of *mtCOL* genes in tobacco failed to induce the expression of *mtFT* (Wong et al., 2014). However, the CO/COL family was shown to be conserved across species (Griffiths et al., 2003; Wong et al., 2014) and this was also confirmed in alfalfa by the phylogenetic analyses we conducted (Figure S1). *FT* genes were also shown to be conserved and five of them were characterized in alfalfa and were proven to have functions (*MsFTa1* in particular*)* in flowering time control, quality of the forage, fibers and protein content (Lorenzo et al., 2020). For these reasons, we believe proposing a microProtein‐based dominant mutation strategy is relevant not only to undercover the function of the CO/COL family in alfalfa, but also to potentially obtain higher biomass and better quality forage.

Such a dominant mutation could be obtained by generating a truncated version of one *CONSTANS‐LIKE* gene leaving the B‐Box domains intact but deleting the CCT‐domain that is needed for DNA‐binding. The synthetic CO‐microProtein could interact with the full‐length CONSTANS proteins, preventing them from binding DNA and thereby delaying flowering. Alfalfa plants expressing such CO‐microProtein could outbreed with wild type plants and the resulting phenotypes of the offspring are expected to be dominant, thereby avoiding the need of homozygosity (Figure 1).

Based on structural variations, the CO/COL family can be subdivided into three main classes: Group I is characterized by having two consecutive B‐box domains near the amino terminus and a CCT (CO, CO‐like, TOC1) domain near the carboxyl end. Group I is further divided into group Ia, containing *CO* as well as *COL1* and *2*, and group Ib that contains *COL3, 4*, and *5*. Group II contains one B‐box domain and includes *COL6, 7, 8*, and *16*. Finally, group III has one conserved and another slightly divergent B‐box domain and includes *COL9 to COL15* (Griffiths et al., 2003). We found many open reading frames in the genome of alfalfa that contain both CCT and B‐box domains. A phylogenetic analysis we conducted using their predicted peptide sequences along with previously classified *COL* homologs in Arabidopsis, soybean and *M. truncatula* grouped them into three classes (Figure S1). The phylogram resembles those performed in *M. truncatula* by Wong et al. (2014) and Ma et al. despite branch support being relatively low in some cases. The genetic distance between orthologs follows clades divergences, increasing confidence in the obtained phylogram. In group Ia, a single ortholog (MsCOL1) can be found (the four alleles a‐b‐c‐d are shown in the phylogenetic tree) in contrast with Arabidopsis where *CONSTANS, COL1*, and *COL2* can be found, supporting the idea that possible *COL* members from this group might have been lost in the Medicago family (Wong et al., 2014). For groups II and III, two and five orthologs were identified, respectively. In cases where all four copies are present in the phylogram, protein sequences were highly similar. Both in alfalfa and *M. truncatula*, QTL mapping approaches have identified significant markers close to a *CONSTANS‐like* gene from group III, which corresponds to *MsCOLh* in the tree in Figure S1. This marker has also been linked with branch length, another trait of high importance for forage quality in alfalfa (Herrmann et al., 2010). It is possible that in alfalfa, *MsCOLh* and related members of group III have acquired roles in flowering induction to compensate for the loss of orthologs from the first group. These members could be promising targets for genetic engineering technologies aiming at the generation of microProtein‐based dominant mutations to delay flowering in alfalfa.

### MicroRNAs, AP2s and SPLs

MicroRNAs are short 21nt single stranded RNA molecules that are processed from larger RNA precursors and known to be involved in the regulation of gene expression at the post‐transcriptional level (Liu et al., 2017). Due to their high evolutionary conservation, sequences of different miRNA classes in alfalfa and their level of homology with other miRNAs in different species can be predicted. These considerations could possibly open up to new approaches in the improvement of alfalfa and specifically in the control of flowering time. MiRNAs, such as *miR156* and *miR172*, were shown to play important roles in flowering time coordination, even in alfalfa. One way of exploiting the CRISPR‐Cas9 technology to explore miRNAs function is to directly mutate the sequence constituting the binding site of miRNAs in respective mRNAs. This method has been shown to work and has been used to verify miRNA targets from different miRNA families. Interestingly, miRNA‐binding site mutations were also used to decipher the *AP2* and *miR172* relationship in flowering‐related phenotypes. This was done in roses, where one of the two alleles of a gene member of the *AP2* family were mutated creating an insertion, leading to a *miR172* resistant gene variant. This insertion disrupting the miRNA binding site correlated with disturbed phenotypes in flower development (Francois et al., 2018). Here, we are proposing a similar approach in alfalfa to create dominant mutations. Considering that miRNA binding site sequences are strongly conserved within gene families, simultaneous editing of multiple *AP2* homologs is a realistic possibility. Thus, in principle and depending on the presence of protospacer adjacent motif (PAM) sequences, one sgRNA may be designed to target all the *miR172* binding sites in the *AP2* family. The *miR172* precursor genes and the mature miRNA sequences are now known in alfalfa and were shown to be identical to the *miR172* mature sequences of *M. truncatula* (Gao et al., 2016). Generation of multiple *miR172*‐resistant *AP2* alleles in alfalfa (Figure 1) would be predicted to result in plants displaying a delay in flowering time, with the resultant other beneficial phenotypes already discussed, in terms of biomass and forage quality.

The delayed flowering phenotype would be expected because of the role *miRNAs* and *AP2s* have in alfalfa, which seems to confirm the function they have in the model plant Arabidopsis. In Arabidopsis, microRNA *miR172* acts as a flowering activator, by negatively regulating *AP2* and other *AP2*‐*like* family members through translational inhibition (Aukerman and Sakai, 2003; O'Maoileidigh et al., 2021). *AP2* genes encode a family of transcription factors that play a central role in the control of flowering time and flower and seed development. *AP2s* act as flowering repressors by negatively regulating the expression of genes such as *SOC1*, *AP1*, and *AG*, which are involved in other flowering pathways.

In alfalfa, 159 *AP2* genes have so far been identified, and functional characterization and expression studies have focused on their role in the abiotic stress response pathways (Jin et al., 2019). Little is known about the role of *AP2*‐mediated flowering control in alfalfa but a similar type of regulation as the one described in Arabidopsis seems plausible. In fact, it has been shown that overexpression of *miR156*, which specifically targets transcription factors belonging to the *SQUAMOSA PROMOTER BINDING PROTEIN‐LIKE* (*SPL*) family, resulted in a decrease of *miR172* precursors (Gao et al., 2016). *SPLs* play multiple critical roles in plant development, ranging from leaf and shoot maturation to the transition from vegetative to reproductive phase and flowering (Wang and Wang, 2015; Wang et al., 2019). Because of their sequence specificity to *SPL*s, *miR156s* can negatively modulate them, thereby controlling major developmental changes in plant development (Wu and Poethig, 2006). Using *M. truncatula* as a template to find *SPL* genes containing complementary regions to *miR156*s (Aung et al., 2015), *SPL* target candidates were amplified in alfalfa. *MsSPL6*, *MsSPL12*, and *MsSPL13* contain miR156‐complementary sites and their transcript levels proportionally decreased as the abundance of *MsmiR156* increased in *miR156* overexpression lines (Aung et al., 2015). Likewise, *MsSPL2*, *MsSPL3*, *MsSPL4*, and *MsSPL9* were down‐regulated significantly in the *miR156* overexpression lines (Gao et al., 2016; Lorenzo et al., 2019). Transgenic alfalfa plants overexpressing *miR156* were shown to exhibit a delay in flowering time, an increase in biomass, higher cellulose levels and reduced lignin content (Aung et al., 2015). *MiRNA156* and *SPLs* are connected to *miR172* and *APs* in a feedback loop and together play crucial roles in the regulation of flowering time. This feedback mechanism changes throughout phases and age of the plant. In this process *AP2* acts as rheostat adjusting the correct balance between *miR156* and *miR172* expression, as documented by *AP2* knockout studies in Arabidopsis (Yant et al., 2010). These findings indicate that respective feedback loop is conserved and that *AP2s* play a role as regulators of flowering in alfalfa as well (Aukerman and Sakai, 2003; Teotia and Tang, 2015; Gao et al., 2016).

Despite some sequence discrepancies among different species, *miR156s* and *miR172s* are highly conserved in the plant kingdom (Wang et al., 2019). The miRNA‐mediated control of phase transition is also conserved across species, in both dicots and monocots, and including perennials and trees. We therefore investigated *miR172* and *AP2* genes as a possible breeding targets in alfalfa. To identify *AP2* genes in alfalfa potentially targeted by *miR172*, we extracted all *AP2* homologs from Arabidopsis. In total, we identified 168 sequences, and phylogenetic analysis grouped five *AP2s* with *miR172*‐complementary region together. We used the collected Arabidopsis sequences to conduct BLAST analyses on the genomes of *M. truncatula*, *G. max* and alfalfa. From these species we obtained a total of 324 hits, using a cut‐off value of E < 1E−10. Five of these were those already identified in Arabidopsis, 81 were *G. max* genes, 29 were *M. truncatula* genes and 209 were alfalfa genes. The 324 genes obtained were subsequently analyzed using the psRNATarget (A Plant Small RNA Target Analysis; [Dai et al., 2018]) online tool to identify potential miR172 targets among the gene sequences derived from the BLASTs. As an input for the analysis the *miR172‐*A sequence of *M. truncatula* was used, shown to be identical to the miRNA172‐a sequence of alfalfa and retrieved from the miRBase Database online (Griffiths‐Jones et al., 2008). We identified 55 gene sequences, of which 28 belong to alfalfa. The predicted amino acid sequences of these genes were used to build the phylogenetic tree that is shown in Figure S2.

## CRISPR‐MEDIATED *CIS*‐ENGINEERING AND REGULATION OF FLOWERING TIME VIA TCPS

CRISPR‐mediated *cis*‐engineering could be another option for obtaining dominant phenotypes that are heritable in the heterozygote state. A bottleneck in *cis*‐engineering is the identification of gene regulatory elements that could be targeted to either increase or decrease the expression of target genes. Considering its roles and its *miR319*‐mediated regulation, the TCP gene family seems to be a promising target for such strategy.

TCP transcription factors were named after the first three members of this family that were characterized (**T** EOSINTE BRANCHED1 [TB1], maize; **C** YCLOIDEA [CYC], snapdragon; **P** ROLIFERATING CELL FACTOR [PCF], rice). TCPs have roles in the regulation of a wide range of plant development processes such as flowering time, nodule development or hormone biosynthesis (Cubas et al., 1999). Like *AP2s*, *TCPs* are also under microRNA control. It was shown that *miR319* (also called *miRJAW*) controls a subset of *TCPs*, referred to as *JAW‐TCPs* (Palatnik et al., 2003; Sarvepalli and Nath, 2018) or MRTCPs (Fang et al., 2021). At least five members have been identified as targets of *mir319* in rice and Arabidopsis, indicating a strong conservation of this mechanism.

We currently have no knowledge on the function of *TCP* genes in alfalfa, but *TCP* genes and the corresponding *miR319* genes can be anticipated to have conserved functions in monocotyledonous and dicotyledonous plants. Studying TCP expression under several conditions as well as *mir319* overexpression in different legumes, including *M. truncatula*, shows that some *JAW‐TCPS* are involved in several developmental programs such as leaf development, flowering time, and nodule formation.

Phylogenetic analyses conducted on the *JAW‐TCP* family in Arabidopsis, soybeans, *M. truncatula* and alfalfa increased the list of possible *JAW‐TCP* members in class II. Among all of the JAW‐TCP genes, the alfalfa genes indicated as *MsTCP4La* and *MsTCP4Lb* in the phylogenetic tree (Figure S3) that cluster together with the *M. truncatula* orthologs *mtTCP3* (XP_013464604.1) and *mtTCP4* (XP_013445507.1) are prominent targets for alfalfa genetic improvement. In Arabidopsis, both *TCP3* and *TCP4* bind the CO promoter increasing its expression. *Tcp4* mutants displayed delayed flowering while overexpression of *atTCP3* and *atTCP4* generated early flowering phenotypes (Kubota et al., 2017). Besides its effect on flowering, *TCP4* is also involved in xylem differentiation through *VND7* regulation (Sun et al., 2017). Overexpression of a *TCP4* version resistant to *mir319* displayed an increase in cell wall thickness and a higher concentration of lignin and cellulose in leaves. In *M. truncatula*, *mtTCP4* and *mtTCP3* were also identified in leaves indicating a possible conservation in roles. Downregulation of *MsTCP4La* and *MsTCP4Lb* could potentially delay flowering while reducing cell wall thickness and lignin concentration, such traits could potentially boost forage quality of alfalfa.

CRISPR‐mediated *cis*‐engineering could prove useful in exploiting the *JAW*/TCP system to induce mutations that would result in dominant phenotypes. In alfalfa gene regulatory elements have not yet been identified in *miR319* genes but recent progress in multiplexed promoter targeting could potentially overcome the bottleneck allowing to either increase or decrease the expression of target genes. In tomato it has been shown that multiplexed targeting of promoters can be used to effectively alter plant growth and development (Rodriguez‐Leal et al., 2017). Such approach in alfalfa may also lead to heritable promoter changes that alter the expression of *miR319* genes causing both a delay in flowering and the production of larger leaves. A parallel strategy could also be to control the expression of *miR319* under different promoters, having tissue specificity. This would allow a more controlled and tailored approach in investigating and generating desired phenotypes.

## CONCLUSIONS

Dominant phenotypes can be achieved by overexpression of genes using conventional transgenic approaches. A drawback of these approaches is the use of herbicide selection markers to select the transgenes and the variability in transgene expression. In addition, the use of viral promoters and non‐host DNA makes these transgenes vulnerable to silencing which can strongly affect trait stability. The induction of dominant mutations using genome‐engineering is a way to bypass aforementioned drawbacks. Some of the strategies proposed in this review are based on microProtein generation by truncation of one gene copy in a group of alleles or in a gene family and on miRNAs‐binding site mutations. Moreover, mutations can be induced in different parental lines simultaneously, increasing the chance of obtaining offspring with the desired phenotype which allows breeders to amplify the seed material more efficiently. Finally, the strategies described here can be used as blueprint for the modification of other crops with complex or polyploid genomes that are obligate outcrossing and adversely affected by inbreeding depression.

## CONFLICTS OF INTEREST

The authors declare they have no competing interests.

## Supporting information

Additional Supporting Information may be found online in the supporting information tab for this article: http://onlinelibrary.wiley.com/doi/10.1111/jipb.13186/suppinfo

**Figure S1.** Phylogenetic tree of CONSTANS and CONSTANS‐like sequences in which members are suggested to control flowering time in *Medicago sativa (*alfalfa)
Homologous genes in *Medicago truncatula* and *Glycine max*, which are closely related to alfalfa, as well as Arabidopsis *thaliana* are also shown. Species origins are highlighted by colored text and circles: red, alfalfa; black; *M. truncatula*; blue, *G. max*; Arabidopsis; green. Using the basic local alignment search tool (BLAST), Arabidopsis sequences were individually used as queries against the *Medicago truncatula* and *Glycine max* protein databases at the Kyoto Encyclopedia of Genes and Genomes (KEGG) webpage. From the results, sequences reporting an *e*‐value ≥ 1E−10 were collected and then blasted to the alfalfa genome (Chen et al., 2020) using the BLAST command line in Ubuntu. In this case as well only sequences reporting an e‐value ≥ 1E−10 were kept. A multiple sequence alignment of the alfalfa sequences was successively conducted using Clustal Omega to check for conserved domains. Only sequences displaying both the BB and CCT domains of CONSTANS were kept. Curated sequences were aligned in MEGA6 using multiple sequence comparison by the log‐expectation (MUSCLE; (Edgar, 2004) and the alignment was subjected to maximum likelihood phylogenetic analysis using RAxML v. 8.2.12 with 1.000 bootstrap iterations and, in addition, Bayesian inference of phylogeny using MrBayes v. 3.2.7 with the parameters: mcmcp nchains = 8; mcmcp temp = 0.05; mcmcp mcmcdiagn = yes; mcmc diagnfreq = 10,000, and run until the average standard deviations of split frequencies was below 0.01. Both analyses were based on a Jones–Taylor–Thornton substitution matrix with inverted gamma distribution and were made using Extreme Science and Engineering Discovery Environment (XSEDE) at the CIPRES ScienceGateway v. 3.3 (Miller et al., 2010). Numbers at nodes refer to bootstrap values above 65. Filled circles at nodes refer to a Bayesian likelihood of 1.00. The alfalfa genes included in the tree and the respective alleles indicated by numbers (1 to 11) and letters (a‐d) and their corresponding accession numbers are: MsCOL1a (MS.gene022048.t1), MsCOL1b (MS.gene75965.t1), MsCOL1c (MS.gene62915.t1), MsCOL1d (MS.gene44781.t1, MsCOL2a (MS.gene33091.t1), MsCOL2b (MS.gene051509.t1), MsCOL2c (MS.gene058459.t1), MsCOL2d (MS.gene016116.t1), MsCOL3a (MS.gene32719.t1), MsCOL3b (MS.gene80166.t1), MsCOL3c (MS.gene80166.t1), MsCOL4a (MS.gene018362.t1), MsCOL4b (MS.gene57909.t1), MsCOL4c (MS.gene035678.t1), MsCOL4d (MS.gene012430.t1), MsCOL5a (MS.gene76302.t1), MsCOL5b (MS.gene065133.t1), MsCOL5c (MS.gene71833.t1), MsCOL5d (MS.gene029402.t1), MsCOL6a(MS.gene04795.t1), MsCOL6b (MS.gene06142.t1), MsCOL6c (MS.gene015721.t1), MsCOL7a (MS.gene44846.t1), MsCOL7b (MS.gene43742.t1), MsCOL7c (MS.gene009935.t1), MsCOL7d(MS.gene88720.t1), MsCOL8a (MS.gene25698.t1), MsCOL8b (MS.gene70471.t1), MsCOL9a (MS.gene029041.t1), MsCOL9b (MS.gene054008.t1), MsCOL10a (MS.gene033677.t1), MsCOL10b (MS.gene72598.t1), MsCOL10c (MS.gene54974.t1), MsCOL11a (MS.gene23011.t1), MsCOL11b (MS.gene006986.t1).
**Figure S2.** Phylogenetic tree of APETALA2‐like sequences in which members are suggested to control flower development in *Medicago sativa (*alfalfa)
Genomic sequences of AP2 homologs in Arabidopsis were collected from NCBI and TAIR and aligned using Clustal Omega. The miR172 Arabidopsis sequences were obtained from “miRbase: the microRNA database” (Griffiths‐Jones et al., 2008). The Plant Small RNA Target Analysis (psRNATarget, (Dai et al., 2018) online tool was used to identify miRNA172 binding sites in the collected sequences. Five Arabidopsis sequences shown to have miRNA172 were used to conduct BLAST analyses on the genomes of *M. truncatula*, *Glycine max*, using KEGG‐Blast. The collected sequences from these species were then blasted to the alfalfa genome. A total of 324 hits was obtained: the five Arabidopsis ones, 81 were *G. max* genes, 29 were *M. truncatula* genes, and 209 were alfalfa genes. The 324 genes obtained were analyzed using the psRNATarget (A Plant Small RNA Target Analysis) online tool to identify miR172 targets. The result was 55 gene sequences, of which 28 alfalfa ones. The sequences were aligned and upon inspection 12 alfalfa sequences were removed, as they only showed a partial alignment and were shown not to belong to the AP2 family, but instead appeared to belong to the Transmembrane 9 superfamily member 8. (MS.gene32702.t1, MS.gene42664.t1, MS.gene80184.t1, MS.gene80181.t1, MS.gene38082.t1, MS.gene020823.t1, MS.gene031691.t1, MS.gene047830.t1, MS.gene003964.t1, MS.gene70874.t1, MS.gene29698.t1, MS.gene56969.t1), resulting in a total of 43 genes. Phylogenetic analysis was essentially as described in the legend to Figure 2. The alfalfa genes included in the tree and the respective alleles indicated by numbers (1 to 11) and letters (a–d) and their corresponding accession numbers are: MsAP2La (MS.gene041052.t1), MsAP2Lb (MS.gene56970.t1), MsAP2Lc (MS.gene010316.t1), MsAP2Ld (MS.gene011791.t1), MsAP2Le (MS.gene56806.t1), MsAP2Lf (MS.gene99769.t1), MsAP2Lg (MS.gene049839.t1), MsAP2Lh (MS.gene65262.t1), MsAP2Li (MS.gene004139.t1), MsAP2Lj (MS.gene08567.t1), MsAP2Lk (MS.gene20030.t1), MsAP2Ll(MS.gene20233.t1), MsAP2Lm (MS.gene09800.t1), MsAP2Ln (MS.gene007473.t1), MsAP2Lo (MS.gene20029.t1), MsAP2Lp (MS.gene22472.t1).
**Figure S3.** Phylogenetic tree of TCP transcription factor‐like sequences in *Medicago sativa* (alfalfa) in which members are suggested to control leaf development and branching
TCPs homologs in Arabidopsis were collected from NCBI and TAIR and used to conduct BLAST analyses on *Medicago truncatula and Glycine max* using the KEGG‐Blast database. The collected sequences from the three species were then blasted to the alfalfa genome. A preliminary phylogenetic analysis was made using all collected sequences. In this analysis, TCPs potentially targeted by miR319s were identified and the clades containing these sequences and a closely related clade were used for making the final tree. Phylogenetic analysis was essentially as described in the legend to Figure 2. The alfalfa genes included in the tree and the respective alleles indicated by numbers (1–11) and letters (a–d) and their corresponding accession numbers are: MsTCPL1a (MS.gene074319.t1), MsTCPL1b (MS.gene053291.t1), MsTCPL1c (MS.gene070930.t1), MsTCPL1d (MS.gene95781.t1), MsTCPL10a (MS.gene031628.t1), MsTCPL10b (MS.gene045511.t1), MsTCPL10c (MS.gene73844.t1), MsTCPL10d (MS.gene045512.t1), MsTCPL10e (MS.gene006670.t1), MsTCP4La (MS.gene059738.t1), MsTCP4Lb (MS.gene060651.t1), MsTCP4Lc(MS.gene028844.t1), MsTCP4Ld (MS.gene54881.t1), MsTCP4Le (MS.gene31403.t1), MsTCP4Lf (MS.gene043478.t1), MsTCP5La (MS.gene93507.t1), MsTCP5Lb (MS.gene83823.t1), MsTCP5Lc (MS.gene79398.t1), MsTCP5Ld (MS.gene28232.t1), MsTCP2La (MS.gene023326.t1), MsTCP2Lb (MS.gene34909.t1), MsTCP2Lc (MS.gene08299.t1).
