# Supplementary material for: Controlling flowering of Medicago sativa (alfalfa) by inducing dominant mutations
Source: J Integr Plant Biol. 2022 Jan 18;64(2):205–14. doi: 10.1111/jipb.13186 (PMC9303315; doi:10.1111/jipb.13186)
Supplement: Supplementary file 1 — Figure S1. Phylogenetic tree of CONSTANS and CONSTANS‐like sequences in which members are suggested to control flowering time in Medicago sativa (alfalfa) Homologous genes in Medicago truncatula and Glycine max, which are closely related to alfalfa, as well as Arabidopsis thaliana are also shown. Species origins are highlighted by colored text and circles: red, alfalfa; black; M. truncatula; blue, G. max; Arabidopsis; green. Using the basic local alignment search tool (BLAST), Arabidopsis sequences were individually used as queries against the Medicago truncatula and Glycine max protein databases at the Kyoto Encyclopedia of Genes and Genomes (KEGG) webpage. From the results, sequences reporting an e‐value ≥ 1E−10 were collected and then blasted to the alfalfa genome (Chen et al., 2020) using the BLAST command line in Ubuntu. In this case as well only sequences reporting an e‐value ≥ 1E−10 were kept. A multiple sequence alignment of the alfalfa sequences was successively conducted using Clustal Omega to check for conserved domains. Only sequences displaying both the BB and CCT domains of CONSTANS were kept. Curated sequences were aligned in MEGA6 using multiple sequence comparison by the log‐expectation (MUSCLE; (Edgar, 2004) and the alignment was subjected to maximum likelihood phylogenetic analysis using RAxML v. 8.2.12 with 1.000 bootstrap iterations and, in addition, Bayesian inference of phylogeny using MrBayes v. 3.2.7 with the parameters: mcmcp nchains = 8; mcmcp temp = 0.05; mcmcp mcmcdiagn = yes; mcmc diagnfreq = 10,000, and run until the average standard deviations of split frequencies was below 0.01. Both analyses were based on a Jones–Taylor–Thornton substitution matrix with inverted gamma distribution and were made using Extreme Science and Engineering Discovery Environment (XSEDE) at the CIPRES ScienceGateway v. 3.3 (Miller et al., 2010). Numbers at nodes refer to bootstrap values above 65. Filled circles at nodes refer to a Bayesian likelihood of 1.00 [file JIPB-64-205-s001.pdf]

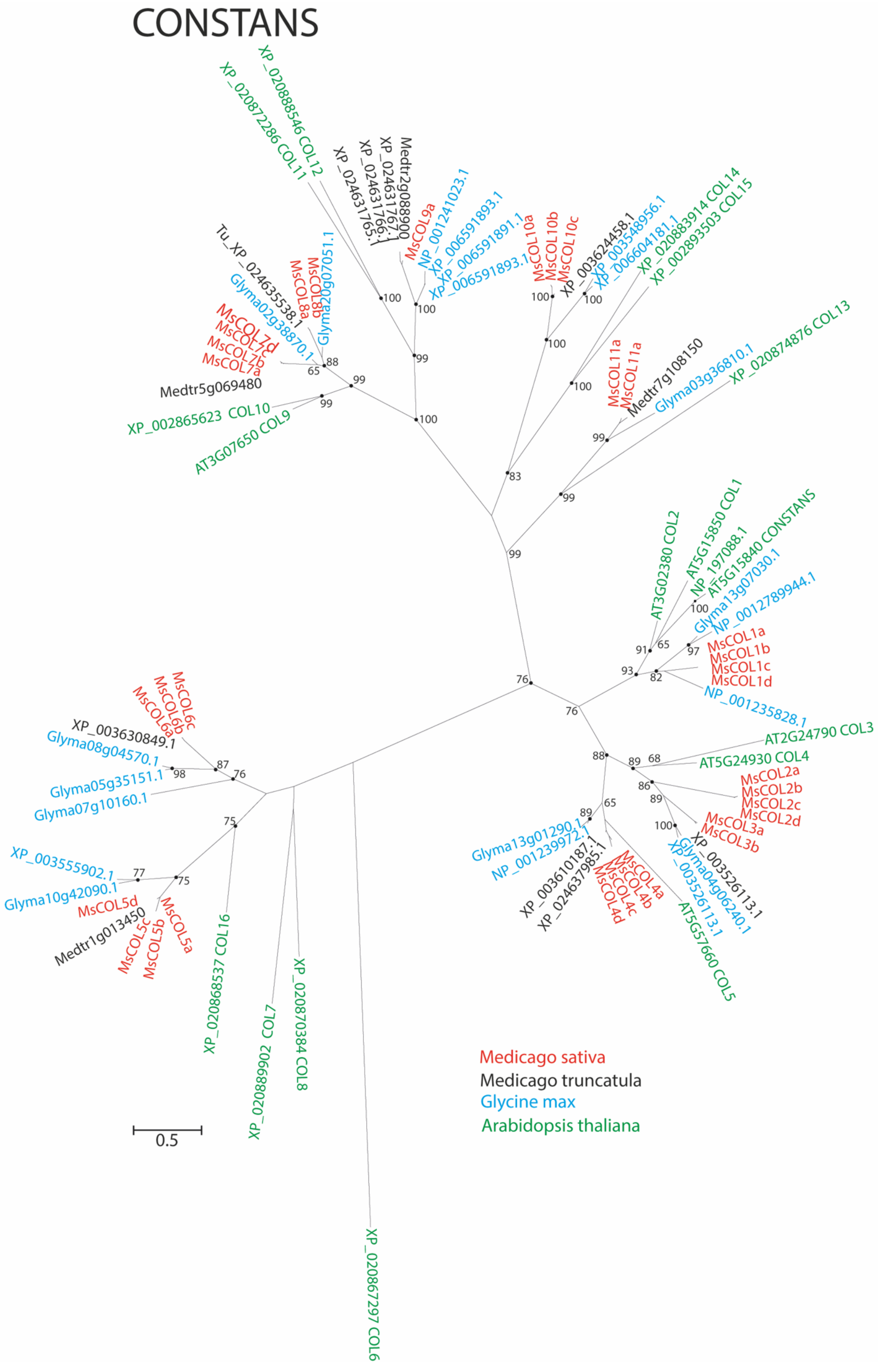
CONSTANS
XP_020888546 COL12
XP_020872286 COL11
Medtr2g088900
XP_024631767.1
XP_024631766.1
XP_024631765.1
MsCOL9a
NP_001241023.1
XP_006591893.1
XP_006591891.1
XP_006591893.1
MsCOL10a
MsCOL10b
MsCOL10c
XP_003624458.1
XP_003548956.1
XP_006604181.1
XP_020883914 COL14
XP_002893503 COL15
XP_020874876 COL13
Glyma03g36810.1
Medtr7g108150
MsCOL11a
MsCOL11a
Tu_XP_024635538.1
Glyma02g38870.1
MsCOL8a
MsCOL8b
Glyma20g07051.1
MsCOL7d
MsCOL7c
MsCOL7b
MsCOL7a
Medtr5g069480
XP_002865623 COL10
AT3G07650 COL9
AT3G02380 COL2
AT5G15850 COL1
NP_197088.1
AT5G15840 CONSTANS
Glyma13g07030.1
NP_001278944.1
MsCOL1a
MsCOL1b
MsCOL1c
MsCOL1d
NP_001235828.1
AT2G24790 COL3
AT5G24930 COL4
MsCOL2a
MsCOL2b
MsCOL2c
MsCOL2d
MsCOL3a
MsCOL3b
XP_003526113.1
Glyma04g06240.1
XP_003526113.1
AT5G57660 COL5
MsCOL4a
MsCOL4b
MsCOL4c
MsCOL4d
XP_024637985.1
XP_003610187.1
NP_001239972.1
Glyma13g01290.1
MsCOL6c
MsCOL6b
MsCOL6a
XP_003630849.1
Glyma08g04570.1
Glyma05g35151.1
Glyma07g10160.1
XP_003555902.1
Glyma10g42090.1
MsCOL5d
Medtr1g013450
MsCOL5c
MsCOL5b
MsCOL5a
XP_020868537 COL16
XP_020889902 COL7
XP_020870384 COL8
XP_020867297 COL6
Medicago sativa
Medicago truncatula
Glycine max
Arabidopsis thaliana
0.5

Figure S1. Phylogenetic tree of CONSTANS and CONSTANS-like sequences in which members are suggested to control flowering time in *M. sativa* (alfalfa)**.** Homologous genes in *Medicago truncatula* and *Glycine ma*x, which are closely related to alfalfa, as well as *Arabidopsis thaliana* are also shown. Species origins are highlighted by coloured text and circles: red, alfalfa;black; *M. truncatula*; blue, *G. max*; Arabidopsis; green. Using the basic local alignment search tool (BLAST), Arabidopsis sequences were individually used as queries against the *Medicago truncatula* and *Glycine max* protein databases at the Kyoto Encyclopedia of Genes and Genomes (KEGG) webpage. From the results, sequences reporting an e-value >= 1E-10 were collected and then blasted to the alfalfa genome (Chen et al. 2020) using the BLAST command line in Ubuntu. In this case as well only sequences reporting an e-value >= 1E-10 were kept. A multiple sequence alignment of the alfalfa sequences was successively conducted using Clustal Omega to check for conserved domains. Only sequences displaying both the BB and CCT domains of CONSTANS were kept. Curated sequences were aligned in MEGA6 using multiple sequence comparison by the log-expectation (MUSCLE; Edgar 2004) and the alignment was subjected to maximum likelihood phylogenetic analysis using RAxML v. 8.2.12 with 1.000 bootstrap iterations and, in addition, Bayesian inference of phylogeny using MrBayes v. 3.2.7 with the parameters: mcmcp nchains=8; mcmcp temp=0.05; mcmcp mcmcdiagn=yes; mcmc diagnfreq=10000, and run until the average standard deviations of split frequencies was below 0,01. Both analyses were based on a Jones-Taylor-Thornton substitution matrix with inverted gamma distribution, and were made using Extreme Science and Engineering Discovery Environment (XSEDE) at the CIPRES ScienceGateway v. 3.3 (Miller et al. 2010). Numbers at nodes refer to bootstrap values above 65. Filled circles at nodes refer to a Bayesian likelihood of 1.00. The alfalfa genes included in the tree and the respective alleles indicated by numbers (1 to 11) and letters (a-d) and their corresponding accession numbers are: MsCOL1a (MS.gene022048.t1), MsCOL1b (MS.gene75965.t1), MsCOL1c (MS.gene62915.t1), MsCOL1d (MS.gene44781.t1, MsCOL2a (MS.gene33091.t1), MsCOL2b (MS.gene051509.t1), MsCOL2c (MS.gene058459.t1), MsCOL2d (MS.gene016116.t1), MsCOL3a (MS.gene32719.t1), MsCOL3b (MS.gene80166.t1), MsCOL3c (MS.gene80166.t1), MsCOL4a (MS.gene018362.t1), MsCOL4b (MS.gene57909.t1), MsCOL4c (MS.gene035678.t1), MsCOL4d (MS.gene012430.t1), MsCOL5a (MS.gene76302.t1), MsCOL5b (MS.gene065133.t1), MsCOL5c (MS.gene71833.t1), MsCOL5d (MS.gene029402.t1), MsCOL6a(MS.gene04795.t1), MsCOL6b (MS.gene06142.t1), MsCOL6c (MS.gene015721.t1), MsCOL7a (MS.gene44846.t1), MsCOL7b (MS.gene43742.t1), MsCOL7c (MS.gene009935.t1), MsCOL7d(MS.gene88720.t1), MsCOL8a (MS.gene25698.t1), MsCOL8b (MS.gene70471.t1), MsCOL9a (MS.gene029041.t1), MsCOL9b (MS.gene054008.t1), MsCOL10a (MS.gene033677.t1), MsCOL10b (MS.gene72598.t1), MsCOL10c (MS.gene54974.t1), MsCOL11a (MS.gene23011.t1), MsCOL11b (MS.gene006986.t1).

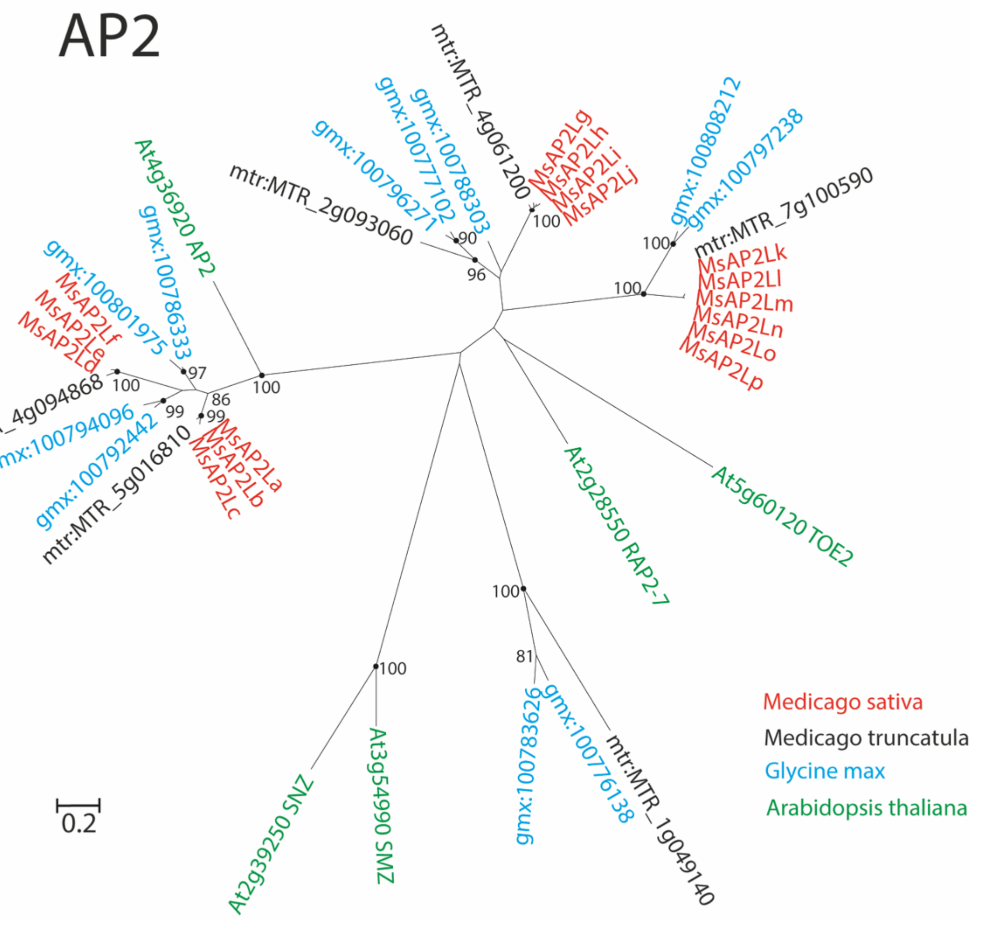

Figure S2. Phylogenetic tree of APETALA2-like sequences in which members are suggested to control flower development in *M. sativa (*alfalfa).

Genomic sequences of AP2 homologs in Arabidopsis were collected from NCBI and TAIR and aligned using Clustal Omega. The miR172 Arabidopsis sequences were obtained from ”miRbase: the microRNA database” (Griffiths-Jones et al. 2008). The Plant Small RNA Target Analysis (psRNATarget, Dai et al. 2018)online tool was used to identify miRNA172 binding sites in the collected sequences. Five Arabidopsis sequences shown to have miRNA172 were used to conduct BLAST analyses on the genomes of *Medicago truncatula*, *Glycine max*, using KEGG-Blast. The collected sequences from these species were then blasted to the alfalfa genome. A total of 324 hits was obtained: the 5 Arabidopsis ones, 81 were *Glycine max* genes, 29 were *M. truncatula* genes, and 209 were alfalfa genes. The 324 genes obtained were analysed using the psRNATarget (A Plant Small RNA Target Analysis) online tool to identify miR172 targets. The result was 55 gene sequences, of which 28 alfalfa ones. The sequences were aligned and upon inspection 12 alfalfa sequences were removed, as they only showed a partial alignment and were shown not to belong to the AP2 family, but instead appeared to

belong to the Transmembrane 9 superfamily member 8. (MS.gene32702.t1, MS.gene42664.t1, MS.gene80184.t1, MS.gene80181.t1, MS.gene38082.t1, MS.gene020823.t1, MS.gene031691.t1, MS.gene047830.t1, MS.gene003964.t1, MS.gene70874.t1, MS.gene29698.t1, MS.gene56969.t1), resulting in a total of 43 genes. Phylogenetic analysis was essentially as described in the legend to Figure 2. The alfalfa genes included in the tree and the respective alleles indicated by numbers (1 to 11) and letters (a-d) and their corresponding accession numbers are: MsAP2La (MS.gene041052.t1), MsAP2Lb (MS.gene56970.t1), MsAP2Lc (MS.gene010316.t1), MsAP2Ld (MS.gene011791.t1), MsAP2Le (MS.gene56806.t1), MsAP2Lf (MS.gene99769.t1), MsAP2Lg (MS.gene049839.t1), MsAP2Lh (MS.gene65262.t1), MsAP2Li (MS.gene004139.t1), MsAP2Lj (MS.gene08567.t1), MsAP2Lk (MS.gene20030.t1), MsAP2Ll(MS.gene20233.t1), MsAP2Lm (MS.gene09800.t1), MsAP2Ln (MS.gene007473.t1), MsAP2Lo (MS.gene20029.t1), MsAP2Lp (MS.gene22472.t1).

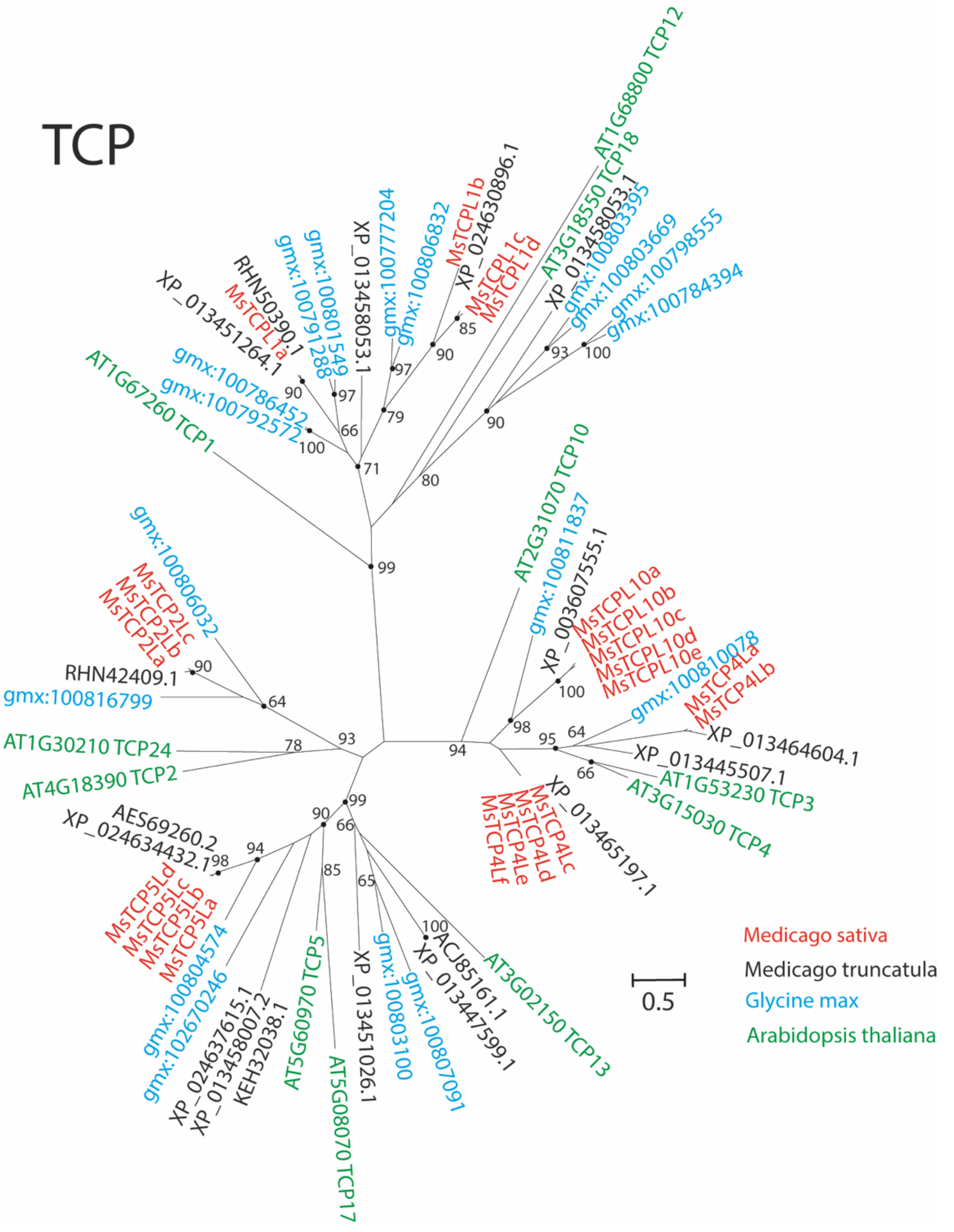

Figure S3. Phylogenetic tree of TCP transcription factor-like sequences in *M. sativa* (alfalfa) **in which members are suggested to control leaf development and branching**.
TCPs homologs in Arabidopsis were collected from NCBI and TAIR and used to conduct BLAST analyses on *M. truncatula and G. max* using the KEGG-Blast database. The collected sequences from the three species were then blasted to the alfalfa genome. A preliminary phylogenetic analysis was made using all collected sequences. In this analysis, TCPs potentially targeted by miR319s were identified and the clades containing these sequnces and a closly related clade where used for making the final tree. Phylogenetic analysis was essentially as described in the legend to Figure 2. The alfalfa genes included in the tree and the respective alleles indicated by numbers (1 to 11) and letters (a-d)

and their corresponding accession numbers are: MsTCPL1a (MS.gene074319.t1), MsTCPL1b (MS.gene053291.t1), MsTCPL1c (MS.gene070930.t1), MsTCPL1d (MS.gene95781.t1), MsTCPL10a (MS.gene031628.t1), MsTCPL10b (MS.gene045511.t1), MsTCPL10c (MS.gene73844.t1), MsTCPL10d (MS.gene045512.t1), MsTCPL10e (MS.gene006670.t1), MsTCP4La (MS.gene059738.t1), MsTCP4Lb (MS.gene060651.t1), MsTCP4Lc (MS.gene028844.t1), MsTCP4Ld (MS.gene54881.t1), MsTCP4Le (MS.gene31403.t1), MsTCP4Lf (MS.gene043478.t1), MsTCP5La (MS.gene93507.t1), MsTCP5Lb (MS.gene83823.t1), MsTCP5Lc (MS.gene79398.t1), MsTCP5Ld (MS.gene28232.t1), MsTCP2La (MS.gene023326.t1), MsTCP2Lb (MS.gene34909.t1), MsTCP2Lc (MS.gene08299.t1).
